# Unusual presentation of paratesticular leiomyoma with prominent vascular features: clinical, surgical, and histological insights

**DOI:** 10.1093/jscr/rjag418

**Published:** 2026-06-18

**Authors:** Mohannad N AbuHaweeleh, Salvatore Rabito

**Affiliations:** College of Medicine, QU Health, Qatar University, PO 2713, Doha, Qatar; Department of Urology, Ambulatory Care Center, Hamad Medical Corporation, PO 3050, Doha, Qatar; Department of Urology, Mater Olbia Hospital, Olbia, Sardinia, Italy

**Keywords:** paratesticular leiomyoma, scrotal mass, angioleiomyoma, benign mesenchymal tumor, testis-sparing surgery

## Abstract

Paratesticular leiomyomas are rare benign mesenchymal tumors arising from smooth muscle of paratesticular tissues, including vascular walls, dartos muscle, and spermatic cord. Angioleiomyoma represents a vascular-rich subtype. They usually present as slow-growing, painless extratesticular masses in middle-aged men. Imaging helps localization but is not diagnostic while histopathology with immunohistochemistry confirms diagnosis. A 49-year-old motocross athlete presented with progressive scrotal enlargement causing discomfort. Examination showed a large encapsulated mass displacing normal testes. Computed tomography revealed a large heterogeneous paratesticular lesion with neoangiogenesis. The mass was excised and scrotoplasty was performed. Histology showed benign spindle cells, low Ki-67 (~3%), and positivity for desmin and CD34. This case emphasizes recognition and testis-sparing management of paratesticular leiomyoma, ensuring excellent outcomes without need for long-term surveillance.

## Introduction

Paratesticular leiomyomas are uncommon benign mesenchymal neoplasms characterized by the clonal proliferation of mature smooth muscle cells originating from local anatomical structures such as the walls of blood vessels, the dartos muscle, or the muscular components of the spermatic cord [1]. Their histogenesis is attributed to smooth muscle fibers present in the walls of blood vessels, the dartos muscle, and the muscular components of the spermatic cord [1, 2].

A rare histological variant, the angioleiomyoma, is characterized by the presence of prominent vascular channels circumferentially invested by interlacing bundles of smooth muscle [2]. These lesions typically exhibit indolent growth kinetics, are well-circumscribed, and lack infiltrative behavior, features that facilitate their distinction from malignant paratesticular neoplasms [1]. The precise etiopathogenesis remains incompletely elucidated; most cases appear to arise sporadically without identifiable predisposing factors. Malignant transformation into leiomyosarcoma has not been clearly established in the literature. 

Hereby, we present a case of a 49-year-old man with paratesticular leiomyoma, manifesting as progressive scrotal enlargement with associated functional impairment, which was definitively managed through complete surgical excision and scrotal reduction plasty, achieving full symptomatic resolution and optimal postoperative anatomical and functional outcomes.

## Case presentation

A 49-year-old gentleman presented with progressive scrotal enlargement persisting for more than one year, associated with significant discomfort and limitation of mobility. His past medical history was unremarkable, with no prior surgeries, no regular medications, and no significant family history. He was a professional competitive motocross athlete. Physical examination revealed a markedly enlarged, globular, well-encapsulated scrotal mass measuring 20 × 17 × 8 cm (Fig. 1), with a tense-elastic to firm consistency; the testes were not palpable. The mass was non-tender, non-ulcerated, and covered with intact skin, without evidence of inflammation or breakdown, extending inferiorly to the mid-thigh. The clinical presentation raised suspicion for a paratesticular neoplasm, warranting further investigations.

**Figure 1:**
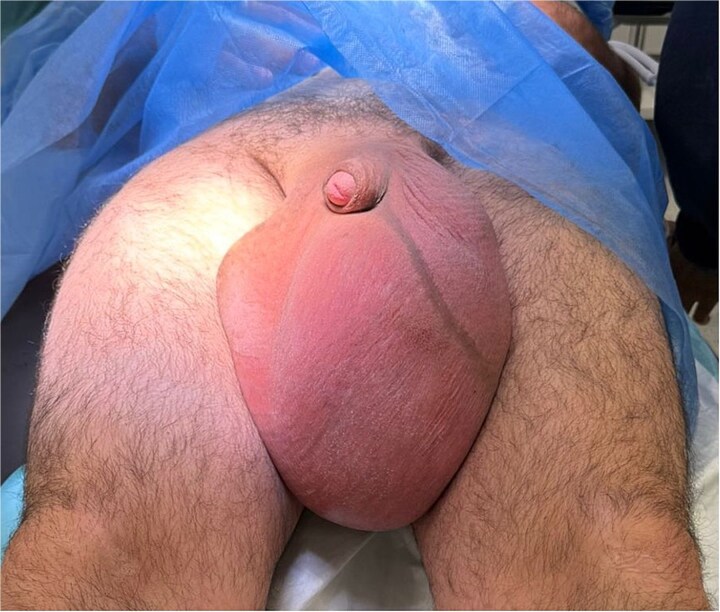
Preoperative examination showing a large, globular, well-encapsulated, non-ulcerated scrotal mass measuring 20 × 17 × 8 cm, covered with intact skin.

## Methods

### Investigations

Ultrasound showed both testes in normal position with homogeneous echotexture and preserved vascularity. A large paratesticular mass displaced them cranially, without testicular or intestinal origin. Computed tomography (CT) revealed a heterogeneous scrotal mass measuring 15.6 × 21.4 × 19.6 cm, with minimal contrast enhancement (Fig. 2). The lesion displaced both testes into the inguinal canals and was associated with marked thickening of scrotal tissues and neoangiogenesis. No lymphadenopathy was seen in inguinal or obturator regions. The bladder and prostate were unremarkable, with no pelvic fluid or bone lesions. Findings suggested a soft tissue scrotal neoplasm, and surgical biopsy was recommended.

**Figure 2:**
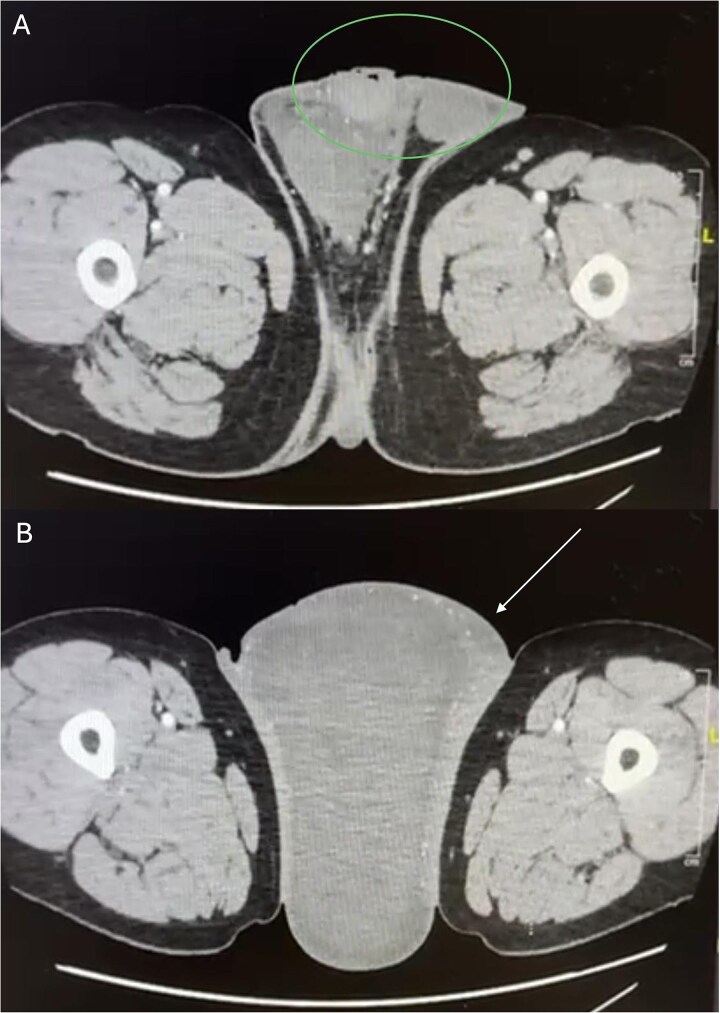
Axial CT) images of the scrotum. (A) Demonstrating a large heterogeneous soft-tissue mass occupying the scrotal sac. The right testis (green circle) is displaced cranially into the inguinal canal by the mass effect. (B) Showing the bulky scrotal mass with marked thickening of the scrotal cutaneous and subcutaneous tissues and multiple small vascular structures suggestive of neoangiogenesis (white arrow).

### Intervention

A midline scrotal incision was made along the raphe, followed by dissection through the dartos fascia. A large median mass was identified and carefully isolated. Hemostasis was achieved by clamping and ligating the neoformed vessels. The mass was excised in its entirety (Fig. 3). Scrotal reduction plasty was subsequently performed. A suction drain was placed, and a compressive dressing was applied with the scrotum maintained in an elevated position. Both the mass and a separate sample of scrotal skin were sent for histopathological examination.

**Figure 3:**
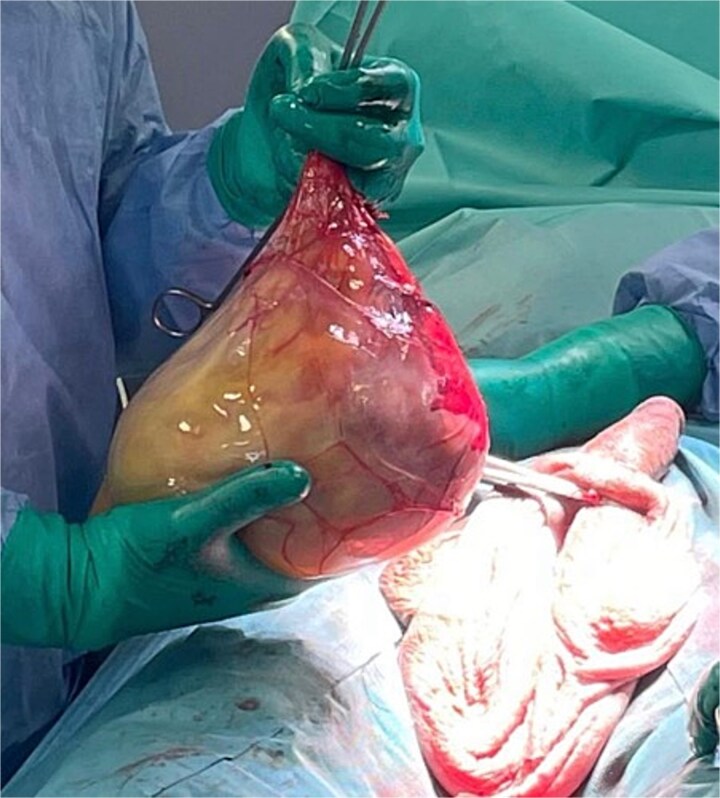
Intraoperative image demonstrating a large, well-encapsulated paratesticular mass following surgical excision. The mass appears smooth and tense with prominent superficial vascularity, consistent with its benign nature.

Histological examination showed a benign mesenchymal neoplasm of moderate cellularity composed of spindle cells without cytological atypia, arranged in concentric and patternless areas within an edematous stroma containing small- to medium-sized vessels. No mitoses or necrosis were identified, and the Ki-67 index was low (~3%). Adjacent skin and subcutaneous tissues showed only edema without significant pathology. Immunohistochemistry was positive for desmin and CD34, supporting smooth muscle differentiation. Findings were consistent with leiomyoma with prominent vascular components, not meeting criteria for angioleiomyoma.

### Prognosis

The patient was hospitalized for two nights, with clean surgical dressings and drain removal on postoperative day two. He was discharged in good condition without additional blood tests. At one week follow up, the wound was clean and well-healed. At 30 days follow up, healing remained excellent, with restoration of normal scrotal anatomy and satisfactory cosmetic outcome. Examination showed an intact surgical site without infection, hematoma, or dehiscence (Fig. 4), with complete symptom resolution. The patient resumed normal social, occupational, and sexual activities.

**Figure 4:**
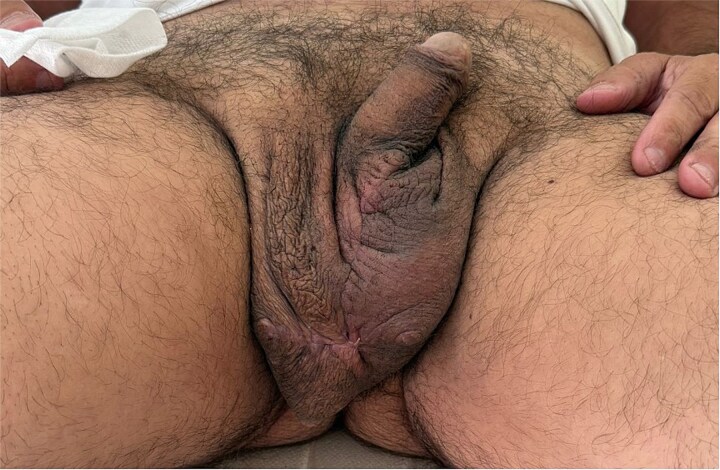
Postoperative clinical examination at 30-day follow-up, showing well-healed scrotal skin with no signs of infection, dehiscence, or recurrence. The scrotum appears symmetrical with preserved anatomical contours, indicating a favorable outcome following excision.

## Discussion

We presented a case of a 49-year-old man with a large paratesticular leiomyoma, successfully treated with complete excision and scrotal reduction plasty, leading to full recovery. Notably, the unusually large size of the mass and its associated mass effect, including displacement of the testis into the inguinal canal, emphasize the clinical significance of this case despite the benign pathology.

The precise incidence of paratesticular leiomyomas remains undefined owing to their extreme rarity; they constitute only a minute proportion of paratesticular neoplasms. Overall, paratesticular tumors represent approximately 7%–10% of all intrascrotal masses in adults, with benign lesions comprising roughly 70% of cases [3]. In the context of our case, the benign presentation, with a well-encapsulated, non-tender mass and absence of systemic symptoms, aligns with the typical features described for benign paratesticular tumors. Among these benign entities, adenomatoid tumors and lipomas are far more prevalent [4], whereas leiomyomas occur infrequently, with most available data derived from isolated case reports or small case series. Bilateral or synchronous presentations are exceptionally rare but have been described [5, 6]. Our patient presented with a unilateral lesion, which is consistent with the majority of reported cases. Limited larger-scale analyses provide additional context; for example, a hospital-based retrospective review conducted between 2013 and 2020 identified only 16 histologically confirmed cases, underscoring their scarcity [7]. Despite this rarity, the clinical and radiological findings in our patient closely mirror those described in prior reports, particularly the presence of a well-defined mass with significant local mass effect. Conversely, some series have reported a relative frequency of up to 17.7% among paratesticular tumors, indicating that while uncommon, leiomyomas constitute a measurable subset in certain institutional experiences [6, 8].

The mainstay of management for paratesticular leiomyomas is complete surgical excision [8], with a preference for testis-sparing approaches whenever technically feasible [9]. Organ-preserving surgery involves meticulous dissection to remove the tumor in its entirety while preserving adjacent normal parenchyma and surrounding structures, thereby minimizing functional and cosmetic morbidity [9]. In our case, a testis-sparing approach was successfully performed despite the large tumor size, as careful intraoperative dissection allowed separation of the mass from the testicular structures. Radical orchiectomy is generally reserved for cases in which preoperative imaging, intraoperative assessment, or frozen section analysis cannot reliably exclude malignancy [8], or when the lesion is densely adherent to the testicular parenchyma, precluding safe separation [8]. Preoperative imaging in our patient supported a benign etiology, and intraoperative findings further confirmed the feasibility of organ preservation, thereby avoiding unnecessary orchiectomy. Intraoperative frozen section evaluation can be invaluable in guiding the surgical strategy, enabling confirmation of the benign nature of the lesion and avoidance of unnecessary orchiectomy [9, 10]. Although frozen section was not utilized in this case, the clear surgical planes and benign characteristics were sufficient to guide management.

The prognosis following complete excision is uniformly favorable. Local recurrence is exceedingly uncommon, reflecting the encapsulated, non-infiltrative growth pattern of these tumors and the adequacy of surgical margins typically achieved. This is consistent with our patient’s outcome, as complete excision was achieved with no evidence of recurrence during follow-up. Long-term follow-up is not routinely required in the absence of histological atypia, although some clinicians advocate for periodic surveillance in select cases, particularly when complete margin clearance is uncertain. Given the benign histopathological findings and complete resection in our case, the likelihood of recurrence is expected to be minimal.

## Conclusion

Paratesticular leiomyomas, though rare, should be considered in slow-growing, well-circumscribed extratesticular masses in middle-aged men. Imaging aids localization but diagnosis relies on histopathology and immunohistochemistry. Testis-sparing complete excision is the preferred treatment, providing excellent outcomes and avoiding unnecessary orchiectomy. Awareness of this entity helps prevent overtreatment and ensures accurate, curative management with minimal morbidity.

## Data Availability

Data sharing not applicable to this article as no datasets were generated or analyzed during the current study.

## References

[1] Hermi A, Saadi A, Mokadem S et al. Paratesticular leiomyoma: a case report of a rare entity. Urol Case Rep 2023;47:102380. 10.1016/j.eucr.2023.102380 36941869 PMC10023848

[2] Rafailidis V, Huang DY, Sidhu PS. Paratesticular lesions: aetiology and appearances on ultrasound. Andrology 2021;9:1383–94. 10.1111/andr.13021 33864338

[3] Galosi AB, Scarpelli M, Mazzucchelli R et al. Adult primary paratesticular mesenchymal tumors with emphasis on a case presentation and discussion of spermatic cord leiomyosarcoma. Diagn Pathol 2014;9:90. 10.1186/1746-1596-9-90 24885500 PMC4039061

[4] Khandeparkar SG, Pinto RG. Histopathological Spectrum of tumor and tumor-like lesions of the paratestis in a tertiary care hospital. Oman Med J 2015;30:461–8. 10.5001/omj.2015.90 26674546 PMC4678445

[5] Ishikawa A, Uraoka N, Shibata J et al. A case of bilateral synchronous paratesticular leiomyoma. Case Rep Oncol 2023;16:88–93. 10.1159/000528821 36820216 PMC9938400

[6] Fernandez A, Krishnamoorthy S, Muralitharan S et al. Bilateral synchronous paratesticular leiomyoma - a rare entity. J Clin Diagn Res 2017;11:PD05–6. 10.7860/JCDR/2017/25109.9518 PMC5449853 28571207

[7] Xue N, Chen Y, Wang G et al. Ultrasound and color Doppler flow imaging of paratesticular leiomyoma. Adv Ultrasound Diagn Ther 2022;6:119–21. 10.37015/AUDT.2021.210022

[8] Irsayanto D, Azmi YA, Rahman IA et al. Paratesticular leiomyoma mimicking an inguinal hernia: a rare case report and literature review. Int J Surg Case Rep 2022;98:107580. 10.1016/j.ijscr.2022.107580 36380540 PMC9468378

[9] Haritha A, SB A, Murthy NR. Testis-sparing approach in paratesticular leiomyoma: a rare case navigating from diagnosis to targeted treatment planning. Cureus 2024;16:e75234. 10.7759/cureus.75234 39759736 PMC11700563

[10] Arslan A, Ulus S, İnce Ü et al. A rare case of paratesticular leiomyoma in a child. Turk J Urol 2019;45:154–6. 10.5152/tud.2018.28813 30875294 PMC6368035

